# PD-L1 reverses depigmentation in Pmel-1 vitiligo mice by increasing the abundance of Tregs in the skin

**DOI:** 10.1038/s41598-018-19407-w

**Published:** 2018-01-25

**Authors:** Xiao Miao, Rong Xu, Bin Fan, Jie Chen, Xin Li, Weiwei Mao, Shengyuan Hua, Bin Li

**Affiliations:** 1 Shanghai University of Traditional Chinese Medicine, Shanghai, 201203 China; 2 Department of Dermatology, Yueyang Hospital of Integrated Traditional Chinese and Western Medicine, Shanghai University of Traditional Chinese Medicine, Shanghai, 200437 China

## Abstract

Programmed cell death 1 ligand 1 (PD-L1) is a ligand of programmed cell death 1 (PD-1) that functions as an immune checkpoint by down-regulating immune responses. To determine whether PD-L1 is a therapy target in vitiligo treatment, Pmel-1 vitiligo mice were treated with a PD-L1 fusion protein. Treatment with this fusion protein significantly reversed/suppressed depigmentation development in adult Pmel-1 mice. Mechanistically, enrichment of regulatory T cells (Treg) in the skin was detected after PD-L1 fusion protein treatment in Pmel-1 mice. Furthermore, Tregs abundance was also increased in both the spleen and circulation of Pmel-1 mice treated with PD-L1. These data indicate that PD-L1 protein therapy inhibits the immune response and reverses depigmentation development in Pmel-1 vitiligo mice.

## Introduction

Vitiligo is a common, disfiguring autoimmune disease of the skin and is characterized by gradual skin depigmentation^1^. The etiology of vitiligo remains unclear. However, the critical role of melanocyte-reactive T cells is well recognized. Histological and genetic studies have demonstrated that vitiligo skin is populated with inflammatory lymphocytic infiltrates consisting of both activated CD4^+^ and CD8^+^ T cells^1,2^. Multiple factors, including genetic factors and environmental stimulation, activate the T cell-specific killing of epidermal melanocytes (MC) that ultimately leads to their death^3^. Different Treg activation strategies, such as CCL22 up-regulation^4^ and simvastatin administration^3^, have demonstrated significant treatment effects in reversing depigmentation in h3TA2 and Pmel-1 vitiligo mice. However, despite the current availability of drug treatments, physical therapy, surgical treatments and other methods, clinical efficacy is not satisfactory. New therapeutic strategies should be evaluated and tested for patients with vitiligo.

Programmed cell death 1 (PD-1) is a member of the extended CTLA-4 family and is a receptor that, functioning as an immune checkpoint, down-regulates immune responses^5^. In response to binding by its ligands, programmed cell death 1 ligand 1(PD-L1) (B7-H1 or CD274) and PD-L2 (B7-DC or CD273)^6,7^, PD-1 represses the activation and function of T cells^5^. PD-1 is not usually expressed on resting T cells, but its expression is induced after T cell activation, for example, by ligation of the T cell receptor (TCR)^8,9^. The absence of PD-1 rendered mice resistant to viral infection and suppressed tumor growth and tumor metastasis by reducing the antigen-recognition threshold and increasing the cytotoxic activity of CD8^+^ T cells. PD-1/PD-L1 pathway-targeted therapies are under development in multiple diseases, including different cancers. Keytruda (pembrolizumab or Lambrolizumab; MK-3475) is a humanized monoclonal IgG4 PD-1 antibody and has been approved by the FDA since September 4, 2014 for patients with advanced or unresected melanoma that no longer responds to other drugs.

PD-L1, also known as B7-H1 and CD274, is a transmembrane glycoprotein and a member of the B7 family of immune regulatory molecules with an approximately 65 kDa. Mature human PD-L1 consists of a 220 amino acid (aa) extracellular domain (ECD) with two immunoglobulin-like domains, a 21 aa transmembrane segment, and a 31 aa cytoplasmic domain, in which shares 73% and 74% aa sequence identity with mouse and rat PD-L1, respectively. B7-H1 is expressed on inflammatory-activated immune cells including macrophages, T cells, and B cells, keratinocytes, enothelial and intestinal epithelial cells, as well as a variety of carcinomas and melanoma^10^. The PD-L1-targeted therapy has also been demonstrated to be effective in experimental psoriasis, a chronic inflammatory skin disorder^11^. Overexpression of PD-1 protein was detected in the skin of the Imiquimod (IMQ)-induced murine model of psoriasis^12^, in which inflammation was inhibited by injecting a recombinant PD-L1 protein^11^. Further experiments indicated that severe psoriatic inflammation was induced in PD-1-null mice^12^. Interestingly, the PD-L1 fusion protein is effective in inhibiting T cell activity in skin autoimmunity disease. Thus, we have a strong rationale to test whether the PD-L1 fusion protein could be a new therapeutic strategy in patients with vitiligo.

Here, we tested the treatment effect of PD-L1 fusion protein in Pmel-1 vitiligo mice. We found that PD-L1 fusion protein treatment reversed depigmentation via a markedly increased abundance of Tregs in the skin.

## Results

### Depigmentation is markedly reversed after PD-L1 fusion protein injection

**Figure 1:**
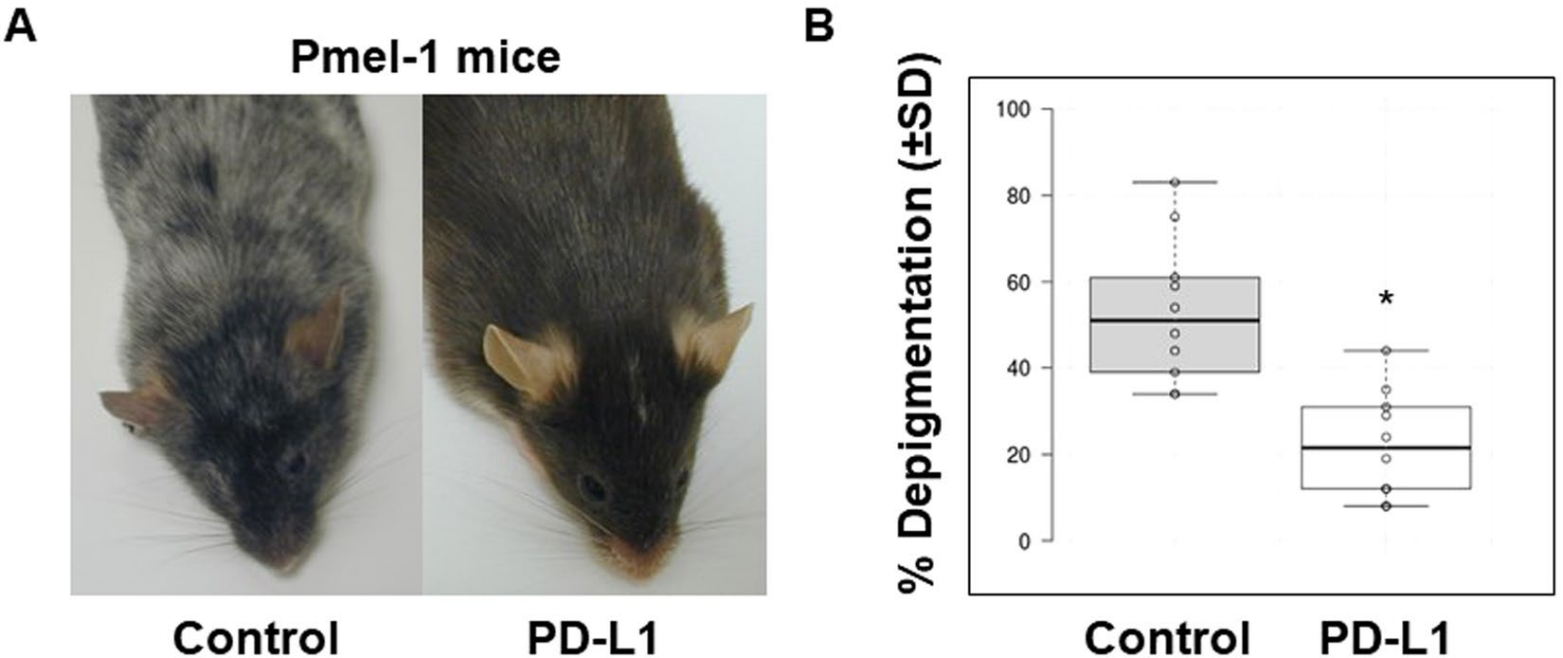
PD-L1 fusion protein treatment significantly reduces pigment degradation in adult Pmel-1 mice. Pmel-1 mice with pigment loss conditions (21 weeks) were treated with mouse PD-L1 fusion protein or IgG control for 6 weeks. Photographs were taken 1 week after the last treatment. (**A**) Pigment loss of representative mice after treatment. (**B**) Quantification of all mice 3 weeks after the last treatment (Control group n = 9 cases, PD-L1 protein treated group n = 8 cases, *p < 0.05).

First, we investigated the therapeutic effect of PD-L1-Fc *in vivo* using Pmel-1 mice, representing a slowly depigmenting model. PD-L1-Fc was administered intraperitoneally at 20 µg/injection 3 times a week for 6 weeks. The recombinant mouse PD-L1/Fc used here is a disulfide-linked homodimeric protein after proteolytic removal of the signal peptide. To produce these recombinant protein, a DNA sequence encoding the extracellular domain (Met 1-Thr 238) of mouse PD-L1 (NP_068693.1) was fused with the C-terminal His-tagged Fc region of human IgG1 at the C-terminus. The PD-L1-Fc has strong affinity with its receptor, PD-1. A linear range of 3.2–400 ng/ml PD-L1-Fc can binds with immobilized recombinant mouse PD-1 at 10 μg/ml (100 μl/well) (http://www.sinobiological.com/PD-L1-B7-H1-CD274-Protein-g-74.html). Depigmentation was observed and quantified once a week for all periods of treatment and for 10 weeks after the last PD-L1 fusion protein injection. Compared with control IgG treatment, PD-L1-Fc treatment significantly reversed depigmentation after the last injection, as represented in Fig. 1A. Depigmentation was quantified in Fig. 1B, and treatment restored 57.8% of the original pigment loss when compared with that of vehicle-treated control animals after the last treatment [22.1 ± 14.6% (n = 8) and 51.4 ± 23.7% (n = 9) depigmentation of regrowing hair, respectively (p = 0.014)]. The significant treatment effects were found until 8 weeks after the last injection. However, the differences between the PD-L1-treated and control groups were no longer significant 9 weeks after the last injection. Fortunately, no significant side effects were observed, including skin carcinogenesis and other skin immune diseases. These results indicate that PD-L1 fusion protein treatment significantly reverses/prevents depigmentation in Pmel-1 mice. These treatment effects were relatively stable but not permanent.

### PD-L1 fusion protein treatment enhances Treg recruitment in the skin

**Figure 2:**
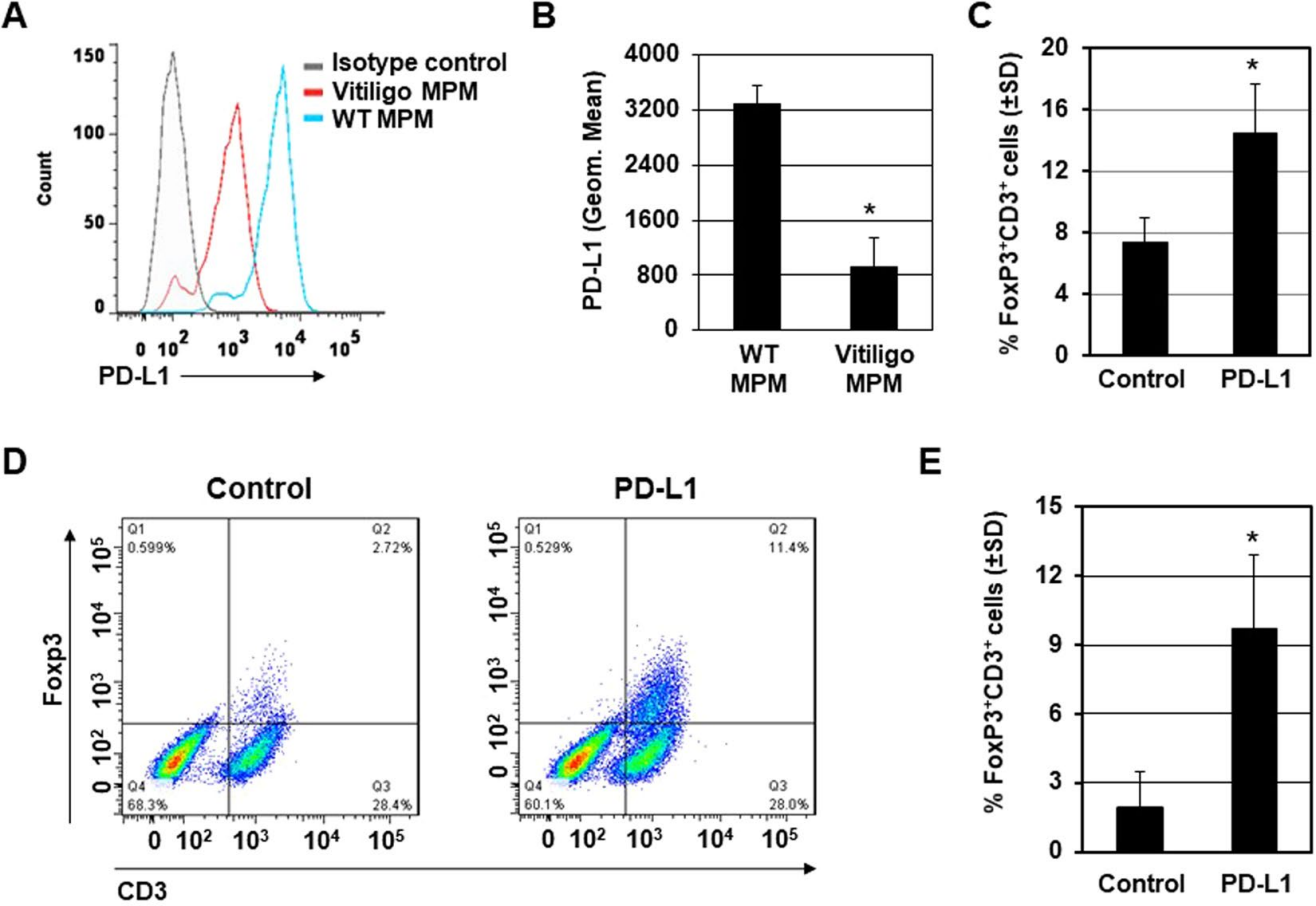
PD-L1 fusion protein treatment regulates T cell enrichment. (**A**) MPM were isolated from Pmel-1 mice. PD-L1 were detected by flow cytometry with specific anti-PD-L1 antibody. (**B**) Quantification of PD-L1 expression in MPM from (**A**). *p < 0.05. (**C**) Expression of FoxP3 and CD3 was evaluated by immunofluorescence staining with specific anti-FoxP3 and anti-CD3 antibodies and then quantified. (**D**) Splenocytes were isolated and assayed for Treg cell population by flow cytometry. Representative flow cytometry images of FoxP3^+^CD3^+^ cell population were showed (Control group n = 6 cases, PD-L1 fusion protein treated group n = 6 cases). (**E**) Quantification of Treg cells population from (**C**) were shown. *p < 0.05.

The development of vitiligo autoimmunity indicates inefficient peripheral tolerance of melanocytes for the host immune system. Regulatory T cells (Tregs), characterized by expression of the forkhead box P3 (FoxP3) signature transcription factor, are crucial for maintaining peripheral tolerance and restraining autoreactive T cells from mediating autoimmune responses^13,14^. Thus, Tregs are major foci in the study of autoimmune pathogenesis of vitiligo^15^. We first detected the expression of PD-L1 protein in isolated epidermal melanocytes in Pmel-1 mice with specific anti-PD-L1 antibodies using flow cytometry. We found that the expression of PD-L1 protein was repressed in Pmel-1 Mouse Primary Melanocytes (MPM) *vs*. WT MPM (Fig. 2A and B).

Next we therefore detected the abundance of Tregs in the skin after PD-L1 fusion protein treatment. Specifically, the skin of aging Pmel-1 mice treated with PD-L1 fusion protein for 6 weeks was harvested 1 week after the final treatment. Treg abundance in the skin of mice treated with PD-L1 fusion protein was detected by immunofluorescence with specific anti-FoxP3 and anti-CD3 antibodies and then quantified. Single staining was also performed with anti-CD25 instead of an anti-FoxP3 antibody. We found that recruitment of Treg was significantly increased by nearly two-fold in PD-L1 fusion protein-treated mouse skin (Fig. 2C). Next, Treg abundance was measured in splenocytes isolated from PD-L1 fusion protein-treated Pmel-1 mice by flow cytometry. We found that FoxP3^+^CD3^+^ Treg cell populations were increased in splenocytes (*P < 0.05) by PD-L1 stimulation. Specifically, average Treg percentages among total T cells in the spleens were 2.7 +/− 1.3% and 11.4 +/− 2.2% in the vehicle group (n = 6) and the PD-L1 group (n = 6), respectively (Fig. 2D and E). These results suggest that PD-L1 fusion protein treatment enhances Treg cell population in vitiligo mice.

### Melanocyte-reactive T cell abundance is repressed after PD-L1 fusion treatment in Pmel-1 mice

**Figure 3:**
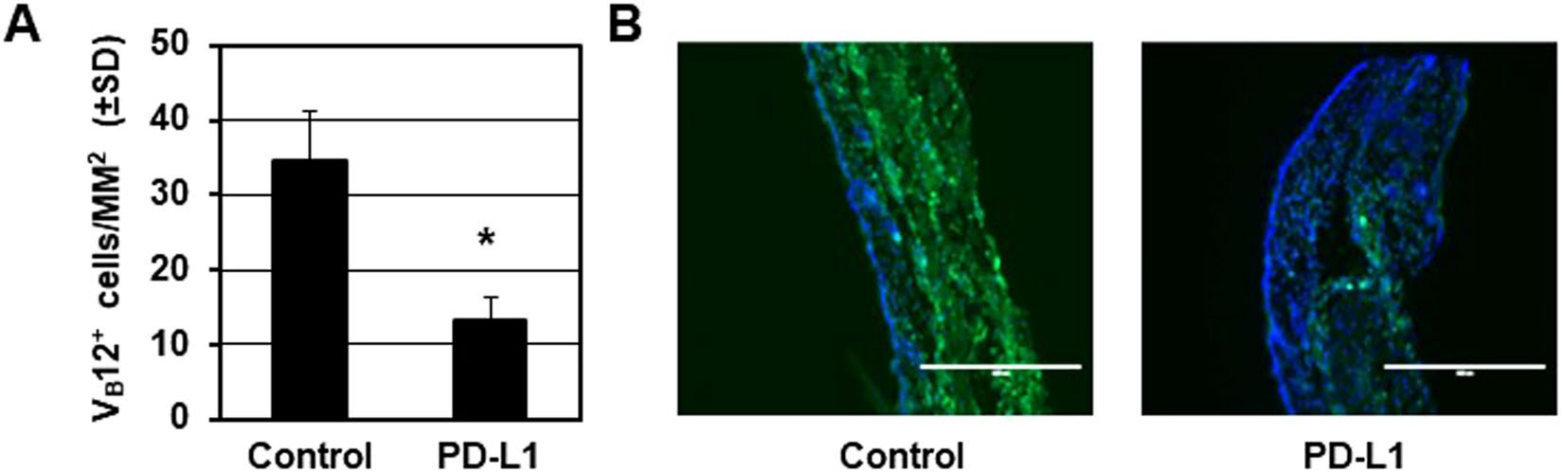
PD-L1 protein reduces melanocyte-specific T cell enrichment in the skin. (**A**) Melanocyte-reactive T cells were detected by immunostaining for the Vβ12 TCR subunit in Pmel-1 mice with PD-L1 protein or control with specific anti-VB12 antibodies and then quantified (Control group = 6 cases, PD-L1 fusion protein = 6 cases, *p < 0.05). Representative staining is presented in (**B**). The scale bar represents 400 μm.

Treg recruitment was detected in the skin. Melanocyte-reactive T cells eliminate melanocytes in the skin to cause depigmentation. Thus, we further detected the effector T cell abundance after PD-L1 fusion protein treatment in Pmel-1 mouse skin. Specifically, melanocyte-reactive T cells were detected by immunostaining for the Vβ12 TCR subunit in Pmel-1 mouse skin. The mice were treated with the PD-L1 fusion protein for 6 weeks and harvested 1 week after the final treatment (n = 6). The numbers of TCR-positive cells were quantified. We found that the abundance of Vβ12-expressing T cells was reduced by 61.4% in the skin of PD-L1 fusion protein-treated mice (13.4 +/− 5.1 cells/mm^2^) compared to the IgG control group (35.1 +/− 7.9 cells/mm^2^) (n = 6, *p* = 0.016) (Fig. 3A and B). These results further indicate that PD-L1 fusion protein treatment induces Treg abundance and suppresses effector T cell recruitment to prevent depigmentation in Pmel-1 mice.

### Enhanced regulatory function is observed in PD-L1 fusion protein-treated mice

**Figure 4:**
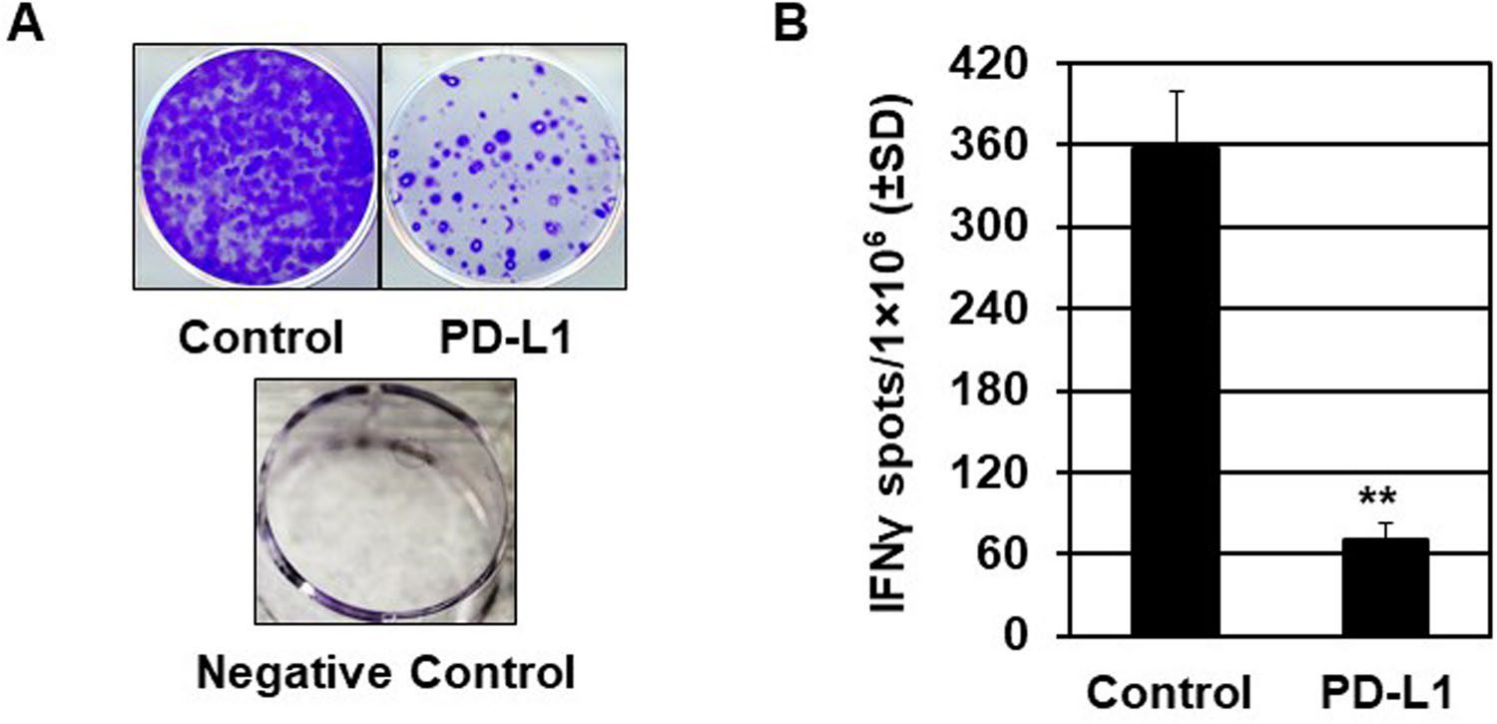
Modulation of immune function in mice treated with PD-L1 protein. Pmel-1 mice were treated with murine PD-L1 fusion protein or IgG control for 6 weeks. One weeks after the final treatment, splenocytes were harvested and incubated for 24 hours with or without peptide. (**A**) The proportion of IFNγ-secreting cells was measured in the presence of 30 μg/ml peptide. (**B**) IFN-γ in the PD-L1 protein treatment group decreased by 77.4% (Control group = 6 cases, PD-L1 fusion protein-treated group = 6 cases, **p < 0.01).

Given the effects of PD-L1 fusion protein-induced Treg abundance and repression of effector T cells in Pmel-1 mouse skin, we further measured the effector T cell functions in treated animals. Splenocytes were isolated from Pmel-1 mice treated with PD-L1 fusion protein or lgG controls for 6 weeks and harvested 1 week after the final treatment (n = 6). Cells were then incubated with cognate peptide prior to quantification of IFN-γ-secreting T cells by ELISPOT. As shown in Fig. 4, T cell activation was detected 61.3 +/− 17.7 spots/well in the in PD-L1-treated group compared to 358.4 +/− 54.3 spots/well in the IgG control group (n = 6, *p* = 0.003) (Fig. 4A and B). These results indicate that PD-L1 fusion protein treatment represses T cell activation and function in Pmel-1 mice.

## Discussion

The pathogenesis of vitiligo involves interactions between intrinsic and extrinsic melanocyte defects, innate immune inflammation and T cell-mediated melanocyte destruction^16^. Useful vitiligo therapeutic strategies should inhibit disease progression and promote repigmentation through simultaneous melanocyte regeneration, proliferation and migration. The center of the effective treatment strategy is to repress melanocyte-reactive T cells^17,18^. Here, we found that PD-L1 fusion protein treatment repressed the abundance of melanocyte-reactive T cells in vitiligo *in vivo*, providing a new potential therapeutic strategy for patients with vitiligo.

The effect of PD-L1 fusion protein treatment on vitiligo was expected. PD-L1 is a ligand of the PD-1 receptor, and PD-1 is a member of the extended CD28/CTLA-4 family of T cell regulators. Both CTLA-4 and PD-1 are receptors that negatively regulate T cell activation. Interestingly, soluble chimeric protein CTLA4Ig (BMS-188667) treatment achieved a 50% or greater sustained improvement in clinical disease activity, with progressively greater effects observed in the highest dosing cohorts in patients with psoriasis^19,20^, another skin autoimmune disease with T cell activation. Furthermore, several independent groups have demonstrated that PD-L1 fusion protein treatment is effective in experimental psoriasis *in vivo*^11,12,21^. In addition, PD-1 antibody treatment resulted in vitiligo development in clinical trials of cancer^22^.

Regulatory T cells (Treg) are characterized by expression of the FoxP3 signature transcription factor, high levels of surface CD25 for IL-2 uptake and other surface molecules and play a pivotal role in maintaining peripheral tolerance and restraining autoreactive T cells from mediating autoimmune responses^13,14^. A critical role of Tregs in vitiligo has been demonstrated by histological studies in humans and in h3TA2 (human TIL derived Tyrosinase TCR transgenic on HLA-A2) vitiligo mouse models^15^. Here, we further demonstrated that Treg repression is also a key factor in Pmel-1 vitiligo development, in which some spontaneous depigmentation of the pelage is observed beyond 20 weeks, and vitiligo develops slowly. Activation of Treg has been recognized as an effective treatment strategy in vitiligo treatment. As a targeted treatment strategy, CCL22 DNA has been demonstrated to promote Treg skin homing to suppress depigmentation^4^. Furthermore, simvastatin treatment also represses effector T cells and activates Treg to reverse depigmentation^3^. Here, we found another targeted treatment strategy in vitiligo treatment, as PD-L1 fusion protein treatment had a similar treatment effect as CCL22 and simvastatin. However, effective treatment with the PD-L1 fusion protein was more stable than either of them. Specifically, we found that the depigmentation suppression effect was observed until 8 weeks after the final treatment with PD-L1 fusion protein compared with <2 weeks and 1 only week for CCL22 DNA and simvastatin treatments, respectively. However, none of them yielded a permanent repigmentation effect. Thus, combination therapies will be required to address all aspects of vitiligo pathogenesis to obtain the greatest treatment efficacy.

## Material and Methods

### Treg assay *in vitro*

Splenocytes isolated from Pmel-1 mice (The Jackson Laboratory, Bar Harbor, ME) were seeded at 1 × 10^6^ cells/well and cultured in RPMI 1640 with 10% FBS and antibiotics (Life Technologies, Grand Island, NY, USA). IFN-γ production was measured directly from cultured splenocytes of treated mice. ELISPOT kits were purchased from MABTECH (Mariemont, OH, USA). Briefly, plates were coated with captured anti-IFN-γ antibodies. IFN-γ-secreting cells were detected using biotinylated anti-cytokine antibodies, followed by horseradish peroxidase-conjugated streptavidin (MABTECH) and 3-amino-9-ethylcarbazole substrate (Sigma-Aldrich, St Louis, MO, USA).

### Mice

Pmel-1 mice with a gp100-reactive transgenic T cell receptor^23^ were obtained from the Jackson Laboratory (Bar Harbor, ME, USA). In an intervention experiment using neutralizing mAb against PD-1, an mPD-L1 fusion protein (50010-M03H-200) was purchased from Sino Biological Inc. Mice were i.p. injected with 200 µg PD-L1 fusion protein or IgG2a on days 1, 3 and 5 of each week for 6 weeks. Mouse fur color and weight were observed once a week. All mouse procedures were approved by the Yueyang Hospital IACUC. All methods were performed in accordance with the relevant guidelines and regulations of the Yueyang Hospital IACUC.

### Histology, immunohistochemistry and immunofluorescent staining

Mouse dorsal skins were formalin-fixed and paraffin-embedded. Hematoxylin and eosin (H&E) staining of paraffin-embedded section (4–5 μm) was conducted according to standard methods. Immunohistochemical staining was performed following the regular procedure with the following antibodies: anti-CD3 (sc-20047), anti-CD4 (sc-7219) and anti-CD8 (sc-7188). Isotype controls (Santa Cruz) were included in each staining. Slides were examined using an Olympus microscope. Quantification of H&E and IHC staining was performed by two experimenters for two sections of two mice per treatment group. Numbers of positive cells were counted for five high power fields (HPFs) and averaged. Immunofluorescent staining was performed following the regular procedure with the following antibodies: anti-CD3 (145-2C11), anti-CD4 (RM4-5), and anti-CD25 (PC61) antibodies (BD Biosciences, San Jose, CA, USA).

### Flow cytometry

Cells were stained in flow cytometry staining buffer (00-4222-26, eBioscience) for 30 minutes. The antibodies used included IFN-γ-PE-Cy7 (25-7319-41, eBioscience). All cells were blocked with Purified Human Fc Receptor Binding Inhibitor (14-9161-71, eBioscience).

### Statistical analysis

The statistical significance of data was determined using a two-tailed unpaired Student’s t-test. The correlation between positive cell numbers of different markers was evaluated by Pearson’s correlation coefficients. Values of *p* < 0.001, *p* < 0.01 and *p* < 0.05 were marked by three, two and one asterisk, respectively.
